# Human Gut Microbiome and Liver Diseases: From Correlation to Causation

**DOI:** 10.3390/microorganisms9051017

**Published:** 2021-05-08

**Authors:** Rui Li, Zhengsheng Mao, Xujun Ye, Tao Zuo

**Affiliations:** 1 Department of Geriatrics, Zhongnan Hospital of Wuhan University, Wuhan 430070, China; wdxjy@whu.edu.cn; 2 Department of Neurology, Wuhan Fourth Hospital, Puai Hospital, Tongji Medical College, Huazhong University of Science and Technology, Wuhan 430074, China; mao_zs@126.com; 3 Guangdong Institute of Gastroenterology, The Sixth Affiliated Hospital of Sun Yat-Sen University, Sun Yat-Sen University, Guangzhou 510000, China

**Keywords:** gut microbiota, chronic liver diseases, metabolites, immune system

## Abstract

The important role of human gut microbiota in liver diseases has long been recognized as dysbiosis and the translocation of certain microbes from the gut to liver. With the development of high-throughput DNA sequencing, the complexity and integrity of the gut microbiome in the whole spectrum of liver diseases is emerging. Specific patterns of gut microbiota have been identified in liver diseases with different causes, including alcoholic, non-alcoholic, and virus induced liver diseases, or even at different stages, ranging from steatohepatitis, fibrosis, cirrhosis, to hepatocellular carcinoma. At the same time, the mechanism of how microbiota contributes to liver diseases goes beyond the traditional function of the gut–liver axis which could lead to liver injury and inflammation. With the application of proteomics, metabolomics, and modern molecular technologies, more microbial metabolites and the complicated interaction of microbiota with host immunity come into our understanding in the liver pathogenesis. Germ-free animal models serve as a workhorse to test the function of microbiota and their derivatives in liver disease models. Here, we review the current evidence on the relationship between gut microbiota and liver diseases, and the mechanisms underlying this phenotype. In addition to original liver diseases, gut microbiota might also affect liver injury in systemic disorders involving multiple organs, as in the case of COVID-19 at a severe state. A better understanding of the gut microbial contribution to liver diseases might help us better benefit from this guest–host relationship and pave the way for novel therapies.

## 1. Introduction

**Figure 1:**
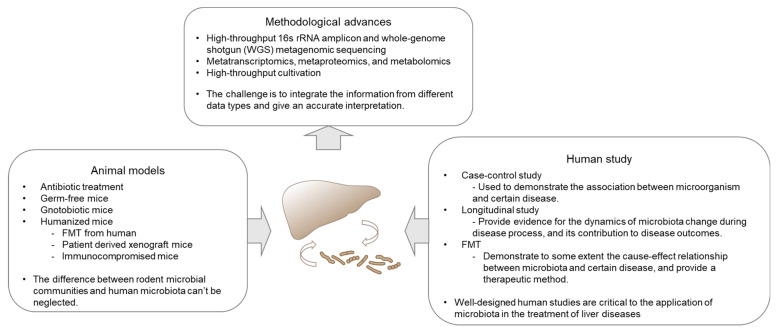
Technology development and the availability of a suite of tools facilitated perception and exploitation of gut microbiota in liver diseases. Multi-omics enables the exploration of the complexity and integrity of the gut microbiome in a spectrum of liver diseases. Mouse models serve as a conductive tool in mechanistic studies, especially the newly developed humanized mice, which resembles human in many aspects, including genetic, immunological and microbiome factors. Human studies reveal the association between microbiota and liver disease, which is the end-resort of microbiota study. Human trials are also critical in final testing of the preventive and therapeutic potential of microbiota in diseases.

Human gut microbiota contains trillions of microbes, contributing to human health and diseases through various routes of mechanism. Recent studies have discovered a myriad of novel functions of gut microbiota linking gut to liver [1,2,3]. With the advancement of high-throughput sequencing, including metagenomics, metatranscriptomics, metabolomics, proteomics, and culturomics, such ever-changing technologies usher the revelation of the tremendously diverse microbiome in humans, in both the intestines and extra-intestinal organs communicating with the gut (Figure 1). Human studies have demonstrated compositional alterations of microbiota in diseases versus health, including those intensively studied in inflammatory bowel disease (IBD), colon cancer, diabetes, and relatively less in liver diseases [4]. However, substantial efforts have been made to unravel the association versus causal relationship between microbiota and disease pathogenesis. In this review, we center on the recent advance in our perception of how gut microbiota contributes to chronic liver disease.

Liver diseases cause ~2 million deaths per year worldwide [5], with cirrhosis and hepatocellular carcinoma as the leading cause of mortality and mobility. Chronic liver disease results from various etiologies, including virus infection, alcoholic liver disease (ALD), and non-alcoholic fatty liver disease (NAFLD). Chronic liver injury can cause liver fibrosis and cirrhosis, with high risk of hepatocellular carcinoma. Nearly 75% of the liver’s blood supply comes from the portal vein, which consists of blood from the intestines and spleen. This special physiological construction of the liver ensures its consistent interaction with the gut-resident microorganisms and their metabolites. Accumulating evidence has demonstrated a sophisticated link between the gut microbiota and the outcomes of chronic liver disease [6,7,8,9]. Gut microbiota has been shown to impact liver diseases through producing metabolic products and modulating host immune responses [10,11,12]. More recently, the alterations of gut microbiota in COVID-19 patients have been studied [13]. Acute liver injury has been reported to be common, around 70%, in patients who test positive for SARS-CoV-2 [14]. The possible contribution of gut microbiota in COVID-19 induced liver injury might be worth further analysis.

Remarkable advances in the understanding of microbiota and liver diseases have been made over the past decades. Building on cutting-edge technologies, we anticipate that our understanding of host-microbial interaction will continue to increase within the coming years. Meanwhile, the advancement in microbial-related diagnostics and therapeutics is envisioned for probing and treating liver diseases [15,16,17]. Herein, we firstly review major clinical and pre-clinical studies to tease the relation between the gut microbiota and various liver diseases. We then explore the evidence for the underlying mechanism of action of the gut microbiota on hepatic function, from two major perspectives, immune calibration and metabolic regulation.

## 2. Clinical Evidence of Microbiota Alterations in Liver Diseases

### 2.1. NAFLD

NAFLD consists of a wide spectrum of liver diseases, ranging from steatosis to nonalcoholic steatohepatitis (NASH), liver fibrosis, and cirrhosis. The pathological process of NASH has been firstly proposed to be the result of “two hits”, which are represented by hepatic steatosis and lipid peroxidation [18]. This theory later evolved into “multiple hits”, comprising signals derived from the gut or the adipose tissue, such as endotoxin, adiponectin, IL-6, and TNFα [19]. Besides dietary and genetic factors, the gut microbiota has been demonstrated as an important novel factor in disease pathogenesis, involving in all aspects of the “multiple hits” [19,20].

The early disease modality leading to NASH is hepatic steatosis, which is viewed as the liver manifestation of metabolic syndrome. The gut microbiota has been linked to a variety of metabolic disorders, with disease-specific signatures [21,22,23,24,25]. Bacterial richness is inversely associated with metabolism-related phenotypes. Individuals with low bacterial richness were at higher odds for insulin resistance, dyslipidemia, and inflammatory phenotype [21]. A decrease in the proportion of *Bacteroides* was observed in obese subjects compared with lean subjects [26], although this finding is not consistent across all studies [27]. In addition, *Bacteroides vulgatus* was reported to impact the serum metabolome and associate with insulin resistance [28], and *Bacteroides* spp. was associated with hepatic steatosis [10]. These data suggest versatile roles of gut microbes in different diseases with similar hepatic pathologies.

More than a decade ago, systematic analysis of gut microbiota was conducted in fatty liver disease [20]. The relation between microbiota and NASH has been discovered in different population-based cohorts. In a pediatric cohort of NASH, Zhu et al. observed a significant increase of the *Enterobacteriaceae* family, accompanied with an increase in blood alcohol concentrations, compared to obese and healthy controls [29]. In view of that *Enterobacteriaceae* could produce ethanol toxic to the liver, this evidence proposed a microbiome-based mechanism to explain the histologic similarity between NASH and alcoholic hepatitis. By contrast, in another adolescent cohort of NAFLD and healthy controls, Mouzaki et al. found no statistical difference in *Escherichia coli* between two groups [30]. Besides, they found a decrease of fecal *Bacteroidetes* in NASH relative to simple steatosis and healthy controls, which is in agreement with the discoveries in obese patients [26]. Echoing these findings, an increased abundance of *Bacteroidetes* and reduced abundance of *Firmicutes* were associated with improvement in hepatic steatosis in a longitudinal study [31]. However, in a group of biopsy-proven NAFLD patients, Boursier et al. observed a higher abundance of *Bacteroides* in NASH and fibrosis patients, compared to those without NASH, suggesting a relation between *Bacteroides* and disease severity [7]. Recently, the high-alcohol-producing *Klebsiella pneumoniae* is found to be associated with NAFLD patients in a Chinese cohort [32]. Again, this data support that an alteration in the gut microbiome contribute to NAFLD through excessing endogenous alcohol production. Other studies, which found no significant difference at the phylum level, documented changes at the class or genus level [33,34]. The discrepancies across these studies might result from small numbers of study subject, intrinsic and extrinsic host factors, including age, gender BMI, environmental, geographical, and dietary factors, and even differences in methodology. Despite these variable findings, the correlation of microbiota and NASH has been well recognized.

Recently, the crosstalk between the microbiome and the human host was comprehensively investigated by integrating metagenome, metabolome and hepatic transcriptome and clinical features [10]. Microbial gene richness decrease was found to be an indicator of deleterious changes. A more complex model was proposed to explain the microbiome–host interplay, involving both metabolism and immune responses. For example, microbial metabolites such as phenylacetic acid (PAA), and microbial products such as Lipopolysaccharide (LPS) have been found to facilitate hepatic lipid accumulation and induce hepatocyte inflammation [10]. Further investigation revealed that *Proteobacteria*, *Actinobacteria* and *Verrucomicrobia* were positively associated with liver steatosis, whereas *Firmicutes* and *Euryarchaeota* were negatively associated with liver steatosis [10]. Overall, the microbial alterations in patients with liver diseases are summarized in Table 1.

Harnessing germ-free (GF) animal models, evidence is accumulating to show a causal effect of microbiota in the pathogenesis of metabolic diseases. Germ-free mice were protected against high fat diet (HFD) induced obesity, and conventionalization of GF mice leads to an increase in body-fat content and insulin resistance [35,36]. Later analysis found obese microbiome could increase energy harvest from the diet. This trait is transmissible through the colonization of germ-free mice with gut microbiota from obese mice (ob/ob mice) [37,38]. Moreover, gut microbiome composition can affect mouse response to HFD, manifested by changes in blood glucose levels and plasma concentrations of pro-inflammatory cytokines. Transplantation of microbiota from HFD mice who have hyperglycemia and higher levels of systemic inflammation can induce the same phenotype in recipient mice [39]. Fecal microbiome from patients with hepatic steatosis could also trigger steatosis in recipient mice [10], which further reinforces the significance of microbiota in the pathogenesis of NAFLD.

**Table 1:** Important studies of human microbiome and liver diseases.

Study	Sample Types	Disease	Method	Major Findings
Mouzaki et al., 2013, *Hepatology* [30]	Stool	NAFLD	Quantitative real-time PCR	The first study assessing the microbiota in adult human NAFLD at different histological stages. Lower percentage of *Bacteroidetes* is associated with NASH.
Jerome et al., 2016, *Hepatology* [7]	Stool	NAFLD	16s sequencing	Gut dysbiosis associates with the severity of NAFLD lesions. *Bacteroides* associated with NASH and *Ruminococcus* with fibrosis.
Loomba et al., 2017, *Cell Metabolism* [17]	Stool	NAFLD	Shotgun sequencing	A gut microbiome signature was identified to predict NAFLD with fibrosis. The abundance of *Firmicutes* and *Proteobacteria* was higher in mild/moderate fibrosis, and advanced fibrosis, separately.
Mutlu et al., 2012, *Am J Physiol Gastrointest Liver Physiol* [40]	Human sigmoid mucosa biopsy	ALD	Multitag-pyrosequencing	The first study showing the association between alcohol consumption and microbiome using non-culture method in human. Lower abundances of *Bacteroidetes* and higher ones of *Proteobacteria* was found in alcoholics.
Dubinkina et al., 2017, *Microbiome* [9]	Stool	ALD	Shotgun sequencing	Alcoholic dependence was inversely associated with *Clostridiales*. The expansion of *Bifidobacterium* and *Lactobacillus* was found in ALD patients, but the species were different between patients with and without cirrhosis.
Liu et al., 2004, *Hepatology* [16]	Stool	Cirrhosis	Quantitative bacteriological Culture	Early evidence of disturbed microecology in cirrhotic patients with MHE, with outgrowth of *Escherichia coli* and *Staphylococcal* species.
Chen et al., 2011, *Hepatology* [41]	Stool	Cirrhosis	454 pyrosequencing 16s	The first major study of microbiome in cirrhosis. Fecal microbial diversity was lower in cirrhotic patients, with higher levels of *Enterobacteriaceae* and *Streptococcaceae*, and lower levels of *Lachnospiraceae*.
Bajaj et al. 2014, *Journal of Hepatology* [42]	Stool	Cirrhosis	Multi-tagged pyrosequencing	The first major study to compare the microbiome in compensated and decompensated cirrhosis. Introduction of CDR, which is associated with disease progression and prognostic.
Qin et al., 2014, *Nature* [43]	Stool	Cirrhosis	Metagenome sequencing	The major study characterized the gut microbiome in liver cirrhosis. Patient-enriched species are of buccal origin, with higher levels of *Proteobacteria* and *Fusobacteria* and fewer levels of *Bacteroides*.
Caussy C et al., 2019, *Nat Commun.* [44]	Stool	Cirrhosis (NAFLD-associated)	16s sequencing	Bacterial signature was discovered to detect NAFLD-cirrhosis based on a prospective twin and family cohort.

### 2.2. ALD

Alcohol has been well demonstrated to be a hepatotoxin, and alcoholic liver disease remains the most prevalent type of chronic liver disease worldwide [45]. The pathogenesis of ALD includes the toxicity of acetaldehyde, hepatic steatosis, and inflammation. Other factors, both genetic and non-genetic, contribute to interindividual variations in disease development. Among them, the gut microbiota plays a critical role in alcohol-induced liver damage. Alcohol abuse leads to bacterial overgrowth, dysbiosis, and gut barrier dysfunction. The subsequent translocation of bacterial products through portal vein stimulates inflammation and metabolic disorders in the liver [46].

Dysbiosis refers to the microbial imbalance in the gut. A pioneer study compared the types and numbers of bacteria in the aspirates from the jejunum of patients with and without alcohol abuse and revealed an increase in the number of microorganisms both anaerobic and aerobic, suggesting a role for bacterial overgrowth in the pathogenesis of chronic alcohol abuse [47]. Later, a study comparing the mucosa-associated colonic microbiome between alcoholics with or without ALD and healthy controls, observed gut microbiome dysbiosis in a subgroup of alcoholics, with lower abundance of *Bacteroidetes* and higher ones of *Proteobacteria* [40]. Altered fecal microbiome was also demonstrated in ALD-related cirrhosis compared to healthy subjects [41,48].

Murine models of alcoholic liver disease also showed an intestinal bacterial overgrowth and enteric dysbiosis in alcohol fed mice [49]. Moreover, compared with conventional mice, germ-free mice showed less liver injury after alcohol consumption [50], corroborating the critical role for gut microbiome in liver disease development. The causal effect of microbiota on ALD has been proposed based on the “leaky gut” theory. The intestinal barrier consists of a mucus layer, physical integrity, and immune defense. Increased intestinal permeability has been described in ALD patients [51]. Through a chromium-51 absorption test in non-intoxicated alcoholic patients, Bjarnason et al. discovered higher intestinal permeability than controls [52]. In alcoholics with chronic liver disease, through measurement of urinary excretion of lactulose and mannitol after oral administration, Keshavarzian et al. observed increased intestinal permeability compared to alcoholics with no liver disease and non alcoholics with liver disease [53]. This “leaky” gut phenotype was also supported by animal models of both acute and chronic ethanol administration [54]. Acute alcohol intake in rats could increase colonic permeability in 24 h and was associated with significant endotoxemia [55]. Ten weeks of alcohol gavage also induced gut leakiness in rats, resulting in endotoxemia and liver injury [56]. Evidence indicates that alcohol-induced gut leakiness and endotoxemia occurs prior to steatohepatitis, acting as a trigger for alcoholic steatohepatitis (ASH) [57].

Further analysis discovered a negative association between alcohol feeding and the expression of certain bactericidal protein in the host, which can be partially reversed by prebiotics treatment. Other manipulations, such as pectin treatment, and fecal microbiota transplantation (FMT), have been shown to be able to prevent alcohol-induced liver injury in mouse models [15]. A similar protective function of microbiota has been observed in ALD patients treated with probiotics and fecal transplantation, which leads to improved liver enzyme levels and better clinical outcomes [58,59]. Oral supplementation of *Akkermansia muciniphila*, which was found to be diminished by ethanol exposure both in mice and humans, can ameliorate experimental ALD and promote intestinal barrier integrity [60]. However, larger clinical studies across different populations with complete evaluation of benefits versus risks are needed before fecal transplantation or other therapeutic regimes built upon manipulation of the gut microbiota can be considered a routine clinical practice in the treatment of ALD.

### 2.3. Cirrhosis

Cirrhosis is the end stage of chronic liver disease, which is characterized by the formation of nodules of regenerative parenchyma, often accompanied by portosystemic shunts. Cirrhosis results from different etiologies, covering all the chronic liver diseases mentioned above, with significant differences between regions, genders, and socioeconomic status [5].

Changes in gut microbiota in patients with liver cirrhosis have been well documented in studies using various approaches. Early studies using bacterial culture found that the microecology was disturbed in cirrhotic patients with minimal hepatic encephalopathy (MHE), characterized by outgrowth of *Escherichia coli* and *Staphylococcal* species. This can be reversed by synbiotic treatment accompanied by a reduction in blood ammonia and amelioration of MHE [16]. A later study using 16S rRNA sequencing observed the distinct gut microbiome in liver cirrhosis, showing that at the phylum level, *Bacteroidetes* was reduced, while *Proteobacteria* and *Fusobacteria* were enriched in the fecal microbiota [41]. Among the bacteria enriched in cirrhosis, *Enterobacteriaceae, Streptococcaceae, and Veillonellaceae* were the most abundant families [41]. A more recent study using quantitative metagenomics revealed that the major change of gut microbiota derives from bloom of oral bacterial species within the gut [43]. Moreover, a combination of 15 biomarkers was used for discrimination of patients with liver cirrhosis from healthy subjects, highlighting the diagnostic potential of microbial markers for liver cirrhosis [43]. These microbial genes, with a high specificity to liver cirrhosis, have no overlap with the markers discovered in type 2 diabetes study [23], which suggests that such microbial features are disease specific.

Other studies have found a correlation between changes of microbiota and severity of disease. Bajaj et al. established a cirrhosis dysbiosis ratio (CDR) based on the ratio of autochthonous to pathogenic taxa, with a low CDR associated with worse disease state [42]. In a longitudinal analysis, comparing patients before and after hepatic encephalopathy (HE) development, CDR was also found to be decreased in patients after occurrence of HE [42]. Loomba et al. showed a shift in microbiome composition during disease progression from mild NAFLD to advanced fibrosis, with an increase in *Proteobacteria* and a decrease in *Firmicutes* at the phylum level. At the species level, *E. rectale* and *B. vulgatus* were the most abundant microorganisms in mild and advanced fibrosis, respectively [17]. They also identified 37 microbial species which distinguish different stages of the disease, suggesting the potential use of microbial markers as a tool in diagnosing and determining the stages of liver disease. Qin et al. found that the severity of liver cirrhosis correlated with the abundance of orally derived species in the gut [43]. In line with these results, studies trying to investigate the effect of gut bacteria on the gut-liver-brain axis by magnetic resonance imaging (MRI), found a positive correlation of *Enterobacteriaceae* and *Streptococcacae* with astrocytic changes. In addition, *Porphyromonadaceae* is associated with changes of neuronal integrity and edema [61].

Commonly used liver cirrhosis/fibrosis mouse models include chemical-based models, which is induced by carbon tetrachloride, thioacetamide or ethanol, diet-based models, mostly methionine-deficient and choline-deficient diet, and surgery-based models, as the common bile duct ligation [62]. Although differences exist between the cause of liver cirrhosis in human and in mouse models, significant change in the gut microbiome were observed in nearly each model [63]. The underlying mechanism of how microbiota effect liver cirrhosis has also been explored by using animal models, which include regulation of gut permeability to release bacterial products and regulation of liver metabolism [64]. However, with the large differences between intestinal microbiota in mice and humans [65], to translate the knowledge gained from mouse studies to human should be very careful [66].

### 2.4. Liver Cancer

The development of liver cancer is a multi-step process, involving factors including genetics, environmental factors, metabolism, and the immune system. Alterations in the microbiota have been reported to contribute to the development of cancer and modulate the efficacy of cancer therapy [67].

In a human study of cirrhotic patients with HCC, the fecal microbiota dysbiosis is characterized by an overgrowth of *E. coli* [68]. In human liver samples, helicobacter was detected in liver carcinoma, while no evidence of helicobacter can be found in patients without malignancy [69]. Studies in animal models have shown that a lack of gut bacteria prevented development of hepatocellular carcinoma (HCC) in different models [6,70]. In HCC mouse model using DEN (diethylnitrosamine) plus CCl_4_ treatment, LPS from the intestinal microbiota contributes to tumor growth by promoting liver cell proliferation, suggesting a role for gram-negative bacteria in tumorgenesis [6]. On the other hand, gram-positive bacterial strains were shown to be increased in mice fed with HFD, and deletion of gram-positive bacteria with vancomycin alleviated the development of HCC induced by DMBA (7,12-dimethylbenz(a) anthracene) and HFD [70].

In addition to the accumulating evidence suggestive of the relation between microbiota and cancer development, the potential role of microbiota in anticancer therapy has been studied [71,72]. In melanoma patients, higher gut microbiome diversity and a relative higher abundance of *Ruminococcaceae* are related with better response to anti-PD-1 immunotherapy [71]. Further study by transplantation (FMT) of stool from responders to anti-PD-1 therapy to germ-free mice showed reduced tumor growth and more importantly, improved responses to anti-PD-1 therapy [71]. In tumor-bearing mice, gut microbiota has been shown to shape the antitumor immune response through translocation into secondary lymphoid organs and thereby stimulating T helper cells [72]. However, no study directly tested the effect of modulating gut microbiota in the treatment of HCC. Targeting the gut–microbiota–liver axis represents an attractive therapeutic option for HCC treatment [73].

## 3. Microorganisms from Other Origins and Kingdoms beyond Fecal Bacteria

Besides fecal microbiota, microbiota from other origins have also been implicated in liver disease, including those from the saliva [74], colonic mucosa [75], sigmoid mucosa [76], and the small intestine [77]. Moreover, evidence suggests that other components of the gut microbiota, including fungi, archaea, and viruses, might play a role in the disease process [78]. Fungal dysbiosis has been illustrated in patients with liver cirrhosis [79]. After antibiotics treatment, fungal diversity was decreased, concomitant with higher prevalence of Candida [79]. A decrease of fungal diversity was also found in alcoholic patients. Overgrowth of Candida was discovered in different stages of alcoholic liver disease, including alcoholic hepatitis and cirrhosis patients [80]. Viruses represent another important collection of microorganisms residing in the gut of humans. Histological severity of NAFLD was associated with a decrease in gut virome diversity [81]. Although high inter-individual variations were found in gut virome composition, several *Lactococcus* phages were found to be decreased in patients with more severe NAFLD [81]. However, an increased fecal virome diversity accompanied by a decreased bacteria diversity was found in patients with alcohol-associated liver disease compared with controls [82]. Others reported only a modest link between fecal phages and liver cirrhosis characteristics [83]. The difference in the change of virome diversity across studies might result from the different types of liver diseases or from different analytical methods. In addition, the gut virome can influence host through interactions with bacteria. When interrogating the phage-bacterial correlations in patients with liver cirrhosis, a higher network complexity was found in controls in contrast to a lower one in cirrhotic patients [83]. Furthermore, a potential therapeutic effect of bacteriophages was shown in ALD [84]. The study showed a correlation between the presence of cytolytic *E. faecalis* and clinical severity of ALD. Then, a therapeutic effect of bacteriophages specifically targeting this bacterium was found in humanized mice colonized with bacteria from ALD patients [84]. However, the involvement and mechanism of gut virome in conjunction with bacteriome in liver disease warrant further investigation.

In addition to live microorganisms, fractions and extracts from bacteria, which are recently recognized as ‘postbiotics’, have been reported to profoundly affect host health [85]. The newest member of the biotics family, postbiotics, refers to bioactive compounds produced by food-grade microorganisms. Although the mechanisms involved in postbiotics bioactivity have not been fully understood, studies have reported various functions of postbiotics, including anti-inflammatory, antihypertensive and antioxidant activity [86,87,88]. For example, Benjamin at al found that whole-cell lysates of the non-commensal bacterium *Methylococcus capsulatus* Bath could improve glucose regulation, diminish hepatic immune infiltration, and change the gut microbiome composition in diet-induced obese mice [89]. Several properties of postbiotics, including their clear chemical structures, stability, and safety dose parameters grant them the potential to become the new generation of health products [90,91]. Although postbiotics is an attractive strategy in the therapeutics of liver diseases, further studies into its mechanism and efficacy are still needed.

## 4. Mechanism of Action of Microbiome in Liver Disease Pathogenesis and Development

**Figure 2:**
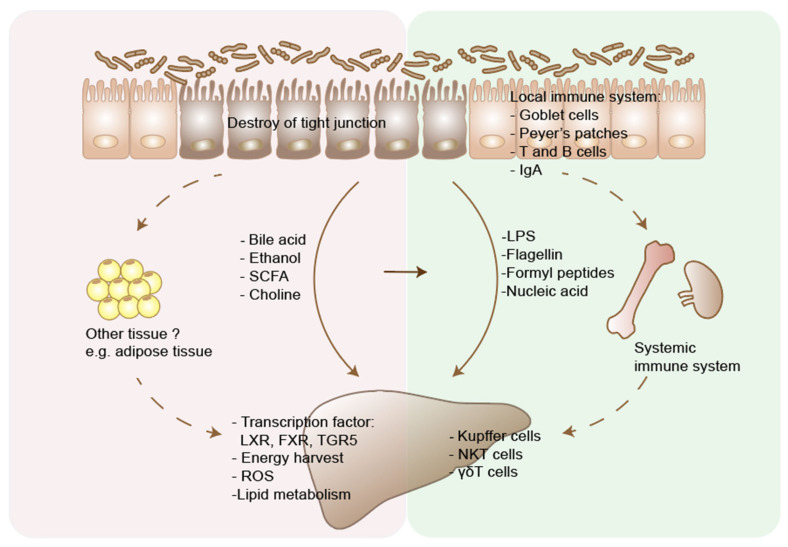
Two main pathways through which gut microbiome affects liver disease. Liver disease results in dysbiosis and intestinal bacterial overgrowth. Disturbed bile acid metabolism affects liver metabolism through regulating the transcription factors, including FXR, LXR, TGR5. Microbiota promote host energy harvest through increasing short chain fatty acids (SCFAs) production. Dietary and microbiota produced ethanol and its metabolites cause hepatocytes damage through generating reactive oxygen species (ROS). Conversion of choline by microbiota causes choline deficiency in host body, which then disturbs lipid metabolism in liver. Gut microbiota could also affect the metabolism of other tissues, e.g., the adipose tissue, and indirectly affect liver diseases, through the chemokines or cytokines interaction. On the other hand, increased intestinal permeability in liver disease leads to translocation of bacteria and microbial products, including LPS, flagellin, formyl peptides and nuclear acids. These pathogens associated molecular patterns (PAMPs) are recognized by pattern recognition receptors (PRRs), such as Toll-like receptors and formyl-peptide receptors, and cause immune cell response in the liver. Microbiota could also modulate host immunity through affecting both local and systemic immune system, which may indirectly affect the progression of liver disease. In addition, microbial metabolites can interact with immune system in liver disease. Primary bile acid could induce NKT cell accumulation in the liver and decrease tumor growth. LXR, liver X receptors; FXR, farnesoid X receptor; G-protein-coupled bile acid receptor 5 (TGR5).

The relationship between dysbiosis of gut microbiome and various liver diseases has been demonstrated in both clinical and preclinical studies. Understanding the mechanism underlying how gut-liver axis functions in relation to liver disease has also been the focus of numerous research studies [1]. Such studies highlight therapeutic approaches of modulating microbiota to treat liver diseases. Although different microbes may contribute to certain types or stages of liver diseases, several common pathways had been discovered. Nutrients and metabolites derived from commensal gut bacterial reach the liver through the portal vein, which can affect hepatocytes metabolism and cause liver damage [1,2,92]. Bacterial and bacterial-derived products, such as lipopolysaccharides, have been implicated in the development of local and systemic immunity, and contribute to liver diseases by interrupting the gut barrier and stimulating liver inflammation [93,94,95,96]. Here we summarize the current knowledge of how gut microbiome affect liver diseases in two aspects, metabolism and immunity (Figure 2). More importantly, bacterial derived metabolites act as crucial modulators of both innate and adaptive immunity [11,97,98]. The microbial metabolites or components implicated in liver diseases are summarized in Table 2.

### 4.1. Metabolism

#### 4.1.1. Bile Acids

Gut microbiota are closely associated with bile acids synthesis and metabolism in the host, contributing to liver diseases by modulating the pool and functionality of bile acids (BAs), while the microbial composition could also be modulated by bile acids.

Bile acids are synthesized from cholesterol by a series of oxidative transformations in the liver and secreted in the form of primary BAs and metabolized in the intestine into secondary BAs by the microbiome. Firstly, microbiota has been found to play a role in the regulation of bile acid synthesis, which is a complex process, catalyzed by a series of enzymes. Study discovered that several of these enzymes, including enzymes involved in bile acid synthesis, CYP7A1, CYP7B1, and CYP27A1, were under microbial regulation [99]. Secondly, microbiota metabolize the BAs in the gut. In humans, the primary bile acids are chenodeoxycholic acid (CDCA) and cholic acid (CA), while in rodents are CA and muricholic acids (MCAs), mainly beta-MCA. Approximately 95% of primary conjugated bile acids are reabsorbed from the intestine by the apical sodium dependent bile acid transporter (ASBT, or known as IBAT), and recirculated back to liver via the portal vein. Through this process, the bile acids form a circle between the liver and the gut, which is called enterohepatic circulation. The microbiota plays an important role in this circulation, affecting BA metabolism. BAs are deconjugated by the bacteria expressing bile salt hydrolase (BSH). Metagenomic analyses reveals a high enrichment of BSH in gut microbiota comparing with other environmental metagenomes, akin to the ones from sea and soil [111]. Deconjugated primary bile acids were metabolized into secondary bile acids deoxycholic acid (DCA) and lithocholic acid (LCA) through 7-dehydroxylation. This process relies on the bacteria with bile acid-inducible (bai) genes, which have been identified in the genera *Clostridium* and *Eubacterium*, both belonging to the *Firmicutes* phylum [92,112]. In the absence of these bacteria, primary conjugated bile acids dominate the bile acid pool [113,114,115]. GF mice have higher proportion of MCAs in the liver, which is a primary bile acid in mice. Apart from DCA and LCA, two major secondary bile acids, ~50 other secondary bile acids exist in human feces, among which isoDCA and isoLCA are the second most abundant secondary bile acids. isoDCA has less detergent capacity and toxicity than DCA and is produced by *E. lentum*, *C. perfringens,* and *R. gnavus*. [116,117,118] The conversion of DCA to isoDCA favors the growth of the keystone genus *Bacteroides* [116].

Bile acids can affect host metabolism through acting as ligands for a variety of transcription factors, the alteration of which involves in metabolic and hepatic disease, including the FXR, LXR, the TGR5, and the vitamin D receptor [92]. Also, these functions of bile acids are closely related with gut microbiota functionality [92]. The gut microbiota promotes FXR signaling in mice through deconjugating TβMCA [99], a natural antagonist of FXR. The gut microbiota is also necessary for producing secondary bile acids which are ligands for TGR5 [119]. Both FXR and TGR5 act as modulators of various components in glucose, lipid and energy metabolisms. Intrahepatic retention of hydrophobic BAs was increased in a NASH-HCC mouse model, which were closely associated with gut microbiota alterations [120]. Obesity related alterations of gut microbiota increased the levels of DCA in mice. The enterohepatic circulation of DCA was implicated in the development of obesity-associated hepatocellular carcinoma, through provoking senescence-associated secretory phenotype in hepatic stellate cells in mice [70]. DCA and LCA have also been demonstrated as a carcinogen in colon cancer both in mouse models and cell lines [121,122,123]. In contrast, ursodeoxycholic acid (UDCA) protects against colon cancer [124]. Moreover, bile acid could largely affect the composition of gut microbiota. Biliary obstruction leads to bacterial overgrowth and translocation of Gram-negative aerobic population, which may perpetuate systemic sepsis [125]. Data also suggest that dysbiosis in cirrhotic patients might be partially due to low bile acid input [126]. In summary, change in microbiota can affect liver disease through the regulation of bile acid metabolism.

#### 4.1.2. Short Chain Fatty Acids

Short Chain Fatty Acids (SCFAs), including acetic, propionic, and butyric acid, are produced by intestinal microbiota through fermentation of undigested carbohydrates. These SCFAs affect host immunity and metabolism in various ways [127]. Gut microbiome, which consume dietary carbohydrate, affect synthesis of SCFAs and contribute to liver homeostasis [128].

Butyrate is an energy source for enterocytes and plays central metabolic roles in maintaining the function of the intestinal barrier [129,130]. A reduction in butyrate is associated with decrease of intestinal tight junction and increased permeability [131]. Supplementation of tributyrin, a glycerol ester of butyrate, reduced intestinal permeability and ameliorated liver injury in an alcohol-induced liver disease mouse model [131]. Besides, studies found that SCFAs can regulate energy harvest and expenditure in the liver, peripheral adipose tissue and muscle [103,132,133]. Previous data found an association between obesity and enrichment of genes involved in carbohydrate fermentation [134]. In contrast, several animal studies found that oral administration of SCFAs reduced body weight in obese mice, mainly through increasing energy expenditure [103,135]. In humans, an increased content of butyrate-producing bacteria was found in recipients with metabolic syndrome receiving intestinal microbiota transplantation from lean donors [136]. And this increase of butyrate-producing bacteria is associated with an improve in insulin sensitivity. The mechanism of how SCFAs regulate energy harvest could be related to the G-protein coupled receptors (GPCRs) of gut enteroendocrine L cells. Activation of these receptors could contribute to the secretion of satiety hormones PYY and GLP-1 [137]. However, the delay in gut transit might also enable increased nutrient absorption. Thus, further human studies on the metabolic function of SCFA producing bacteria should be performed.

#### 4.1.3. Ethanol

Alcohol is absorbed by stomach and small intestine and reaches the liver via the portal vein. Alcohol and its metabolites cause damage to hepatocytes through generation of free radicals, especially reactive oxygen species (ROS). Besides the direct effect on the liver, ethanol and its metabolites also destroy tight junction of intestine cell wall [138]. Intestinal microbiome has been discovered to mediate the increase in intestinal permeability and liver damage induced by ethanol.

Gut microbiota and enterocytes metabolize the ethanol into acetaldehyde and acetate [139,140]. Plasma concentrations of ethanol were increased, while luminal concentrations of acetaldehyde were decreased in antibiotic-treated rats than in controls, after acute alcohol intake. In vitro analysis discovered that acetaldehyde but not ethanol could significantly increase intestine permeability measured by dextran flux, suggesting the importance of the microbiome relied on the oxidation of ethanol into acetaldehyde in gut barrier alteration [55]. Acetaldehyde has also been demonstrated to disrupt intestinal tight junction and increase permeability in epithelial cell line Caco-2 cells [141]. With disturbed gut barrier, pathogen associated molecular pattern (PAMPs) reached the liver and contributed to liver damage and inflammation [142]. Sterilization with antibiotics prevented liver injury in rats following long-term exposure to ethanol [143]. In contrast, greater liver injury and inflammation were observed in germ-free mice after oral consumption of ethanol compared to conventional mice [139]. The discrepancy might be caused by different duration of ethanol exposure. While reducing the intestinal bacterial load with antibiotics attenuated chronic liver inflammation, the complete absence of the microbiota also causes problems in face of acute injury.

In addition to oral consumption, ethanol can also be derived from carbohydrates fermentation by gut microbiota [144]. Similarities of histological features exists between alcoholic and non-alcoholic fatty liver diseases, which suggest that certain common pathogenic processes underpin these two liver disease types. Patients with nonalcoholic fatty liver disease are discovered to have increased luminal and circulation levels of ethanol, with an increase in proteobacteria, which produce alcohol [29]. This provides evidence for non-dietary ethanol produced by intestinal bacteria in the pathogenesis of NASH. Similarly, modest increase in breath ethanol were detected in obese patients compared with lean controls with biopsy proven NASH, who have not drunk alcohol recently [145]. Ob/ob mice that have developed NASH was discovered to have higher early-morning breath alcohol content compared with lean mice [146].

#### 4.1.4. Choline

Choline is an essential nutrient, which is important for cell structure and neurotransmitter synthesis [147]. Mice feed a choline-deficient diet for 4 weeks can induce a NASH-like syndrome and thus be used to model human NASH [148,149]. Choline deficiency has also been correlated with fatty liver in human studies [33,108]. In the liver, choline is metabolized into phosphatidylcholine. Phosphatidylcholine assist the synthesis and excretion of VLDL, and its deficiency leads to hepatic accumulation of triglycerides [107]. In addition, choline can also be processed to trimethylamine (TMA) by intestinal bacteria, which are absorbed through the microvilli and circulated via the portal vein to the liver [150]. In the absence of microbiota, as in germ-free mice and antibiotic-pretreated animals, the amount of urinary TMA was greatly reduced, suggesting an important role for the gut microbiota in choline metabolism [151]. TMA is then converted to trimethylamine N-oxide (TMAO) [152]. An adverse association was found between the plasma TMAO level and the severity of NAFLD in hospital and community-based adults [108]. Besides, increased TMAO was correlated with cardiovascular events both in human and mice [24,153]. Altered intestinal microbiota could stimulate hepatic fat deposition and contribute to liver injury through modulation of dietary choline metabolism [154].

In 129S6 mice, which is susceptible to metabolic diseases, choline metabolism was found as a cause for its disease susceptibility [155]. The bioavailability of choline was reduced in this murine strain as a result of the conversion of choline into methylamines by microbiota. The decrease of circulating plasma choline levels created a phenotype similar to that caused by choline-deficient diets, suggesting an active role of choline metabolism by microbiota in the development of NAFLD [155]. On the other hand, gut microbiota also changed in response to choline deficiency. In individuals fed a choline depletion diet, higher levels of fat accumulation in the liver were associated with the increased levels of *Gammaproteobacteria* and *Erysipelotrichi* in the gut, underscoring the role of microbiota in metabolic disorders [33].

### 4.2. Immunity

#### 4.2.1. The Impact of Commensal Bacterial on Local and Systemic Immunity

Commensal microorganism intestinal colonization is influenced by maternal transfer, mode of delivery, contact and diet [96]. An adult-like intestinal microbiota is established by three years of age and maintained a relatively stable state [156]. However, microbiome can be disturbed in disease states or with administration of antibiotics and medications [157,158,159]. The gut microbiota is involved in the development of local mucosal immunity and regulation of systemic immunity [95,160,161].

The importance of gut microbiota on host mucosal immunity is well corroborated through various studies comparing germ-free mice with conventionally raised mice [162,163]. The intestinal immune system is underdeveloped in germ-free animals. Firstly, germ-free mice have fewer and smaller goblet cells, and thinner mucus layer, which is the first anatomical site of defense against intestinal pathogens [161,163]. Then, germ-free mice have smaller mesenteric lymph nodes and smaller Peyer’s patches [164,165]. Bacteria also helped the genesis of isolated lymphoid follicles (ILFs) in mice and the maturation of ILFs into large B-cell clusters [166]. Finally, T and B cells in the Peyer’s patches, CD4+ lymphocytes in the lamina propra, and IgA-secreting plasma cells are numerically affected by germ-free conditions and some of which can be restored by conventionalization [167,168]. These features render the germ-free animals impaired local immune response to pathogens [169,170,171], and more prone to infections [172].

In addition to mucosal immune response, gut microbiota also plays a vital role in shaping systemic immunity. Early studies demonstrated the role of commensal microbes in the regulation of bone marrow myelopoiesis [95,173,174]. In line with previous studies, recent study reports a global decrease of the proportions and differentiation potential of specific myeloid cell progenitors, granulocyte and/or monocyte progenitors (GMPs) in germ-free mice [175]. Thus, the immune defense against the infection with *Listeria monocytogenes* were decreased in germ-free and oral-antibiotic-treated mice [175].

Lack of commensal bacteria also increase host susceptibility to allergic disease through increasing serum IgE and circulating basophil populations [160]. In addition, a single commensal bacterial species, segmented filamentous bacteria, can drive autoimmune arthritis in germ free animals partially through inducing autoantibodies production [176]. These evidence show the ability of microbiota to shape the global immune system and modulate the host susceptibility to inflammation and infection.

#### 4.2.2. The Impact of Gut Microbiome and Its Derivatives on Innate and Adaptive Immunity

##### Innate Immunity

Microorganisms and their metabolic products are implicated in the homeostasis of the innate immune system and can also be regulated by the host responses [177].

Innate immune system locates at the host-microbiome interface to protect the integrity of gut barrier [177]. The intestinal epithelial cells are equipped with pattern recognition receptors (PRRs), such as Toll like receptors (TLRs) and NOD-like receptors (NLRs), which can sense conserved components of the microbiome. The lack of these innate immune receptors leads to dysbiosis and disrupted gut epithelial barrier, predisposing tissue to inflammation [178,179]. The inflammasome signaling in epithelial cells, which could induce IL-18 and downstream antimicrobial peptides production, is the best-characterized molecular mechanisms for maintaining epithelial integrity [180]. The microbiota-associated metabolite taurine has been found to positively regulate the NLRP6 inflammasome pathway, while histamine and spermine play a negative role in this situation [181]. What is more, SCFAs can activate the NLRP3 inflammasome pathway through binding to GPR43 and GPR109A on epithelial cells and protect against colitis [180]. Besides metabolites, the protozoan *Tritrichomonas musculis* has been found to activate the inflammasome to protect against bacterial infections, at the same time increasing the risk of inflammatory disease [182].

In addition to epithelial cells, microbiota and their metabolites also interact with innate lymphoid cells and other non-classical lymphocytes. First, innate lymphoid cells are a newly discovered arm of the innate immune system, which play critical roles in mucosal immunity [183]. Genome-wide chromatin and transcriptional profiling combined with single-cell transcriptomic analysis described a comprehensive map of ILCs subsets in the intestine and found a critical impact of microbiota on the gene expression of ILCs, which is involved in the cell fate determination [183]. Again, metabolites from the microbiota have been found to regulate ILCs function. Zelante et al. discovered that tryptophan metabolites can induce IL-22 production of ILCs, which in turn regulates the survival of microbial communities, providing resistance to fungus infection [98]. Next, liver-resident γδT cells were identified and demonstrated to be a connection between microbiota and hepatic immune response. Hepatic γδT cells can be activated by microbiota lipid antigens and produce proinflammatory cytokine IL-17A [184]. Finally, NKT cells are enriched in the liver and lies in the middle of the interactions between microbiota and hepatic immunosurveillance. Primary bile acid can increase CXCL16 expression in liver sinusoidal endothelial cells, which induce NKT cell accumulation and decrease liver tumor growth. Colonization of bile acid-metabolizing bacteria reversed both the NKT cell accumulation and its effect on tumor inhibition [11].

##### Adaptive Immunity

Microbiota can influence the adaptive immune system in the gut mucosa, such as differentiation of CD4^+^ T cells and IgA-producing B cells in Peyer’s patches and lamina propria. Imbalances in the gut microbiota triggers immune disorders, which can result in systemic outcomes distant from the site of colonization [185].

IgA is secreted in the gut and serves as an important first-line barrier restricting the access of intestinal antigens to the host. Lack of IgA changes the function of microbes in intestine [186]. On the contrary, microbial stimulation is required for the production of IgA, as demonstrated in the example of segmented filamentous bacterium (SFB) and *Alcaligenes* [164,187]. The production of IgA can be regulated by gut microbiota in both T-cell-dependent and T-cell-independent pathways. T-cell-dependent responses are usually directed against protein antigens [188]. T-cell-independent response is elicited by commensal bacteria and produce polyreactive specificities with low affinity for commensals [189,190], while limited exceptions exist [191]. On the other hand, some bacteria have been found to degrade the secretory component of IgA and IgA itself [192]. IgA coating allows bacteria translocation [193]. An enrichment of IgA-coated *Escherichia coli* was found in Crohn’s disease-associated peripheral spondyloarthritis compared to Crohn’s disease alone. Colonization of this *Escherichia coli* induced T helper 17 cell immunity and lead to more severe colitis and arthritis in mouse models [194].

CD4^+^T cells mainly locate in the lamina propria, which differentiate into Tregs and various T helper cells upon activation by microbiota. Th17 cells and Treg cells are the most extensively studied subsets of T cells in the interaction of microbiota and host diseases [96]. Intestinal microbes, especially SFB, can induce intestinal Th17 cells response [195]. Th17 cells have been found enriched in the intestine of human IBD patients [194]. The transplantation of IBD microbiota into germ-free mice increased the amount of intestinal Th17 cells and Th2 cells, while decreased the number of a specific Treg cells, the ROR γt+ Treg cells [196]. The imbalance between Th17 cells and RORγt+ Treg cells also accounted for disease severity in the colitis mouse model [196]. Next, the number and function of Treg cells can be affected by gut microbiota [197]. For instance, species of 73 *genus* [198], *Bacteroides fragilis* and its polysaccharide A (PSA) [199], promoted Treg cell accumulation and differentiation. What is more, inoculation of *Clostridium* and oral treatment of PSA can lead to resistance or even reversing of experimental colitis in mice [198,199]. Finally, microbiota metabolites, SCFA, have been discovered to regulate the size and function of Treg cell pool and protect against colitis in mouse models [200]. Later, SCFAs was also found to directly promote T cell differentiation into both effector and regulatory T cells [201]. These evidence reveal that microbiota and its metabolites underlie adaptive immune regulation and promote colonic homeostasis and health.

## 5. Gut Microbial Molecules in Liver Immune Modulation

Microbial components translocate through the portal vein into the liver and directly modulate the immune response in liver, thus affecting its pathogenesis, progression, and development. The immune system recognizes pathogen-associated molecular patterns (PAMPs) through pattern recognition receptors (PRRs). LPS is the cell component of gram-negative bacteria, which can increase inflammation, metabolic syndrome, and fibrosis in the liver [202,203]. NASH patients had higher levels of LPS in peripheral circulation and in the liver compared with controls [204]. In high fat diet fed mice, the level of LPS increases by 2–3 folds [202], with an increase of LPS-containing microbiota. On the other hand, LPS infusion alone can increase visceral and subcutaneous fat deposition in mice which is similar to the effect induced by high-fat diet [202]. The number of macrophages in adipose tissue and levels of inflammatory markers were also increased due to LPS infusion [202]. Most of these features, both in LPS and high-fat diet-induced metabolic diseases, were abolished or attenuated in the CD14 mutant mice, which is a main LPS receptor [202].

LPS stimulates Kupffer cells through an essential signaling cascade and thus induces the production of immune regulating cytokines [93]. After integrating with LBP, the LPS-LBP complex binds to CD14 which associates with TLR on the cell surface and stimulates the downstream mitogen-activated protein kinases (MAPK), JNK, p38, and NF-κB pathway. This leads to the transcription of proinflammatory cytokines, including TNF, IL-1, and IL-6. Evidence from animal models and patients support that this signaling pathway is activated in NASH. As mentioned above, mice without CD14 were protected from the development of steatosis after LPS treatment [202]. In MCD-diet fed mice, TLR4 ligand challenge aggravated liver injury and increases proinflammatory cytokine secretion [205].

Other microbial components, flagellin, formyl peptides, and nucleic acid can also regulate immune response and impact liver pathophysiology. Flagellin, the primary structural component of flagella, is a typical pathogen-associated molecular pattern. It can be sensed by TLR5 on the cell surface and signals through MyD88, resulting in the production of inflammatory cytokines and chemokines [206]. Flagellin administration can induce liver injury, which is associated with neutrophils and macrophages accumulation in the liver [207]. Next, human Formyl-peptide receptors FPRs is considered as an PRR, which recognize the peptides cleaved of bacterial [208]. Fprs deficiency exacerbated the severity of infection in mice, which is related with impaired neutrophil recruitment to the liver [209]. This suggests a role of formyl-peptide in the induction of immune response in liver in the early stage of infection. Finally, nucleic acids from bacteria also play a major role in intestinal immune homeostasis. Unmethylated cytosine phosphate guanosine (CpG) dinucleotides limit Treg cell’s suppressive function [210], and DNA derived from conventional gut flora DNA (gfNDA) could also inhibit Treg cell conversion and modulate the Treg/Teff equilibrium [169]. TLR-9 recognizes the CpG containing DNA from bacteria and virus. TLR-9 deficient mice showed reduced liver injury, with dampened hepatic neutrophil infiltration [211]. Through these different mechanisms, bacteria and its components modulate the immune balance of the liver against pathogens and antigens.

## 6. Microbiota in Therapeutics of Liver Diseases

**Figure 3:**
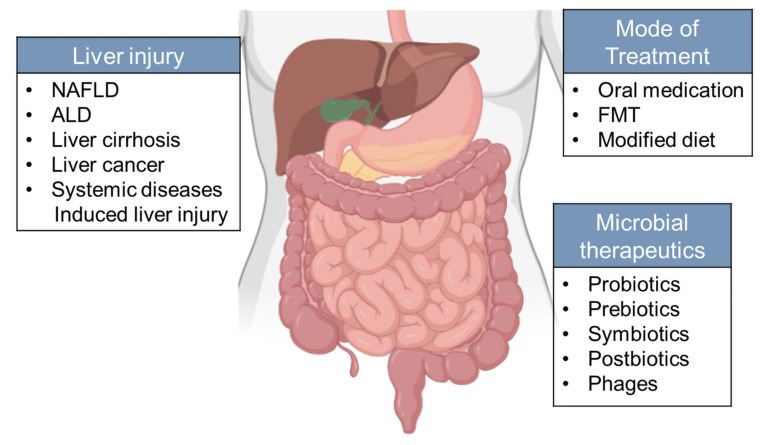
Microbiota-oriented interventions can be applied to different types of chronic liver disease. Strategies aimed at changing gut microbiota include supplementation of prebiotics, probiotics, symbiotics, and postbiotics on the host. However, the therapeutic potential of microorganisms from other kingdoms (phages) remains to be explored. Mode of treatment include oral administration, FMT and modified diet.

Based on the important roles of dysbiosis in the development of liver diseases, various approaches to restructuring the gut microbiota have been tried in the treatment and prevention of liver diseases. (Figure 3).

Treatments of antibiotics [212], prebiotics [213], and probiotics [214,215] have been demonstrated to hinder the development of NAFLD in mouse models. The underlying mechanisms of these approaches cater for protecting against the increased translocation of bacterial endotoxin and the subsequent activation of Kupffer cells and induction of TNF-α. For example, administration of VSL#3, a multistrain cocktail composed of several species of *Lactobacillus* and *Bifidobacteria,* and *Streptococcus thermophilus*, could decrease liver TNF-α levels and limit inflammatory liver damage in rats receiving HFD [216]. *Lactobacillus casei Shirota* treatment attenuated the activation of TLR4 and protected against NAFLD in mouse model of fructose-induced steatosis [215]. Then, oligofructose (OFS) supplementation in NASH patients significantly decreased serum ALT and AST levels compared to placebo in a small-scale pilot study [217], suggesting the potential of prebiotics in the treatment of NASH. Finally, the combination of probiotics and prebiotics also showed positive results in the treatment of NASH [218].

In cirrhosis, the modulation of gut microbiota has also been demonstrated to be a potential therapy in both preclinical experiments and clinical trials. VSL#3 treatment significantly improved the Child–Turcotte–Pugh score and reduced the need for hospitalization in cirrhotic patients compared with placebo-treated controls [219]. In addition, FMT increased gut microbiota diversity and beneficial taxa, and improved the cognition levels in patients with recurrent HE, compared with patients receiving standard of care alone [220]. FMT showed similar protective effects in carbon tetrachloride (CCl_4_)-induced HE in rats [221].

Recently, the potential role of gut microbiota in the chemotherapy and immunotherapy has been highlighted in several studies. The loss of certain commensal bacterial by antibiotics treatment inhibited anti-tumor Th17 cell induction, and thus compromised the efficacy of cyclophosphamide in the treatment of sarcomas in mice [72]. Antibiotics also inhibited the efficacy of anti-CTLA4 immunotherapy in mouse sarcomas, possibly through to the decrease of *B. fragilis* and *B.thetaiotaomicron* which could induce the immune response mediated by Th1 cells [222]. In another mouse model of melanoma, commensal bacterial enhanced the efficiency of programmed cell death protein 1 ligand (PD-L1) therapy, which has been identified to be the effect of *Bifidobacterium* [223]. However, it remains to be studied whether the beneficial anti-tumor properties of gut microbiota can be applied to liver cancer treatment.

In addition to bacteria, dysbiosis of microorganisms from other kingdoms, including fungi, archaea and viruses, and postbiotics were found to be associated with liver diseases [78,79,80]. An antifungal drug has been found to prevent ethanol-induced steatohepatitis in mice [79]. However, whether manipulating the intestinal mycobiome can be an effective strategy for alcohol-related liver disease still need to be tested in patients. Bacteriophages are viruses which can infect and kill bacteria and play a role in preserving the microbiome equilibrium. A recent study found that bacteriophages specifically targeting cytolytic *E. faecalis* can abolish ethanol-induced liver disease in humanized mice [84]. Still, clinical trials are warranted to validate the therapeutic potential of such phage-oriented strategy.

## 7. Potential Impact of Microbiota on Liver Injury in COVID-19 Patients

In December 2019, an outbreak of a novel coronavirus disease, COVID-19, was first reported in China, and then caused a pandemic worldwide. COVID-19 is mainly a respiratory disease, but could also lead to cardiac, kidney, and liver injuries [224]. Although elevations in transaminases are often mild, COVID-19 patients with abnormal liver biochemistries show higher percentages of severe cases [14,225]. In addition, patients with severe COVID-19 are more likely to have liver injury [226]. The mechanism by which the disease-causing agent SARS-CoV-2 affects the liver could be ascribed to direct viral cytotoxicity or immune mediated inflammatory damage [227]. Other factors such as drug-induced liver injury, sepsis, and thrombosis also contribute to the liver damage [227].

Recently, fecal microbiota alterations were studied in COVID-19 patients [13]. Gut dysbiosis existed in COVID-19 patients at the time of hospitalization and persisted after the clearance of the virus. Baseline abundance of the bacteria *Coprobacillus* and *Clostridium* showed significant correlation with COVID-19 severity, while four *Bacteroidetes* inversely correlated with SARS-CoV-2 load over the course of hospitalization [13]. Among them, *Bacteroidetes dorei* have been shown to down-regulate the expression of angiotensin converting enzyme 2 (ACE2) in murine colon [228]. SARS-CoV-2 uses the ACE2 receptor to enter host cells. Thus, this data might propose a protective role of microbiota in SARS-CoV-2 infection through hampering its host entry ability. ACE2 receptor is also presented in biliary and hepatic endothelial cells [229]. However, whether the microbiota or its derivative molecules could affect the infection of SARS-CoV-2 in the liver remains unclear. In addition, *Clostridium* is one of the main bacteria species that deconjugate primary bile acid [112]. Knowing the importance of bile acids in regulating immune response in the liver, the impact of *Clostridium* enrichment in COVID-19 patients on liver injury is worthy of further investigation. Finally, given the mutual interactions discovered between viral hepatitis and the human microbiome, [230] whether the changes in microbiota could lead to liver injury through modulating the immune response to this new virus, SARS-CoV-2, needs further investigation. In addition, dysbiosis of fungi and viruses were discovered in COVID-19 patients [231,232]. Increased proportions of *Aspergillus flavus* were found in COVID-19 patients compared with controls [232]. *Aspergillus flavus* is the fungi that produce aflatoxin, which is a known human carcinogen of HCC. Moreover, the fact that systemic viral infection by SARS-CoV-2 correlates with gut microbiome composition and function [231] could possibly be generalized to other hepatitis virus infected clinical manifestations, as demonstrated by Aly et al. [230]. Given the importance of fungi and viruses in host immune system and their interaction with co-habiting bacteria in the GI tract, their functions in disease course need further in-depth exploration.

As of now, limited data is available on the relation between COVID-19 and microbial dysbiosis. Further studies with larger population should be conducted and more affected organs should be taken into account in exploring the effect of microbiota on this worldwide health crisis.

## 8. Outlook

Based on the large amount of evidence showing the relationship between microbiota and liver diseases, specific and accurate gut microbial contributions to the pathogenesis and therapeutics of liver diseases has become the centerpiece of present studies [233]. With improved computational techniques and experimental designs, a combination of gut microbiota analysis with other clinical examinations will serve as the standard to define liver disease state and to predict disease sensitivity in the near future. In addition to gut microbiota, an intrahepatic bacterial metataxonomic signature has been identified in NAFLD patients, which provide further evidence for the interaction of microbiota in disease manifestation and mechanisms.

Animal models play an indispensable role in mechanistic studies to dissect the causations between microbiota and liver diseases. With more humanized animal models becoming available [234,235], the gap between animal disease models and human patients will be further narrowed down in many respects, including the genetic, immunological, and microbiome features. Nevertheless, well-designed and large-scale clinical trials are needed to effectively translate and apply the findings from bench to bedside.

## Figures and Tables

**Table 2:** Microbial metabolites or components implicated in liver diseases.

Microbial Metabolites or Components	Function of Microbiota	Effects	Mechanisms	References
Bile acids	Microbiota deconjugates primary bile acids and changes the primary/secondary ratio of bile acids.	Effects in the gut	Tauro-conjugated beta-muricholic acids (TβMCA) inhibit FXR-dependent Fgf15 expression in the ileum, which then increase bile acid synthesis in the liver.	[99]
		Effects In the liver	Bile acids activate FXR, vitamin D receptor, and TGR5, and regulate the metabolism of glucose, fatty acid, triglyceride and VLDL.	[100]
		Effects on immune system	Primary bile acids regulate CXCL16 level on liver sinusoidal endothelial cells, which controls the NKT cells accumulation and inhibit liver tumor growth.	[11]
Short chain fatty acids	Microbiota produce short chain fatty acids through fermentation of polysaccharides	Effects in the gut	Butyrates are energy sources for enterocytes and help maintain the integrity of the intestinal barrier.	[101]
			SCFAs act on G-protein receptors (GPCRs) GPR41 and GPR43 on gut enteroendocrine L cells and enhance nutrient absorption.	[102]
		Effects In the liver	SCFAs could increase hepatic lipid oxidation and lower hepatic lipid synthesis.	[103]
		Effects on immune system	Butyrates suppress inflammation through inducing the differentiation of colonic Treg cells.	[104]
Ethanol	Microbiota contribute to the metabolism of ethanol into acetaldehyde and acetate. Microbiota also produce ethanol through fermentation of carbohydrates.	Effects in the gut	Acetaldehyde could significantly increase intestine permeability.	[55]
		Effects In the liver	Alcohol and its metabolites cause damage to hepatocytes through generation of free radicals, which cause oxidative stress.	[105]
		Effects on immune system	Ethanol-induced gut barrier dysfunction and translocation leads to activation of Kupffer cells, infiltrating neutrophils and macrophages, which release proinflammatory cytokines and cause parenchymal cell death.	[106]
Choline	Choline can be processed to trimethylamine (TMA) by intestinal bacteria, which can lead to reduced availability of dietary choline.	Effects In the liver	Choline deficiency inhibits VLDL excretion from the liver and leads to the hepatic accumulation of triglycerides.	[107]
			Trimethylamine N-oxide (TMAO), derived from TMA, is associated with liver damage. However, a causal relationship between the two still needs to be clarified.	[108]
Pathogen-associated molecular patterns, including cell wall components and DNA	LPS is the cell component of gram-negative bacteria. Flagellin, the primary structural component of flagella.	Effects in the gut immune system	Activation of TLRs on intestinal epithelial cells promotes epithelial cell proliferation, secretion of IgA and antimicrobial peptides.	[109]
		Effects In the liver immune system	Activation of TLRs on hepatic Kupffer cells and HSCs leads to inflammation and fibrosis, through inducing a range of cytokines, including IL-1, IL-6 and TNF.	[110]
